# Iron Overload in Histidine-to-Aspartic Acid Substitution at 63 (H63D) Gene Heterozygous Hereditary Hemochromatosis With Erythrocytosis: A Case Report

**DOI:** 10.7759/cureus.76335

**Published:** 2024-12-24

**Authors:** Ishanya Abeyagunawardena, Sinnathurai Mayura, Piyum Samarasingha, Achala Nawinne, Harindra Karunatilake

**Affiliations:** 1 Internal Medicine, National Hospital of Sri Lanka, Colombo, LKA; 2 Hematology, National Hospital of Sri Lanka, Colombo, LKA

**Keywords:** case report, h63d mutation, haemochromatosis, idiopathic erythrocytosis, iron overload

## Abstract

Hereditary hemochromatosis occurs due to genetic mutations, namely, cysteine-to-tyrosine substitution at amino acid 282 (C282Y) and histidine-to-aspartic acid substitution at 63 (H63D) mutations. The role of H63D mutation in hemochromatosis is less clear, and its penetrance is low even in homozygotes. Therefore, iron overload in H63D heterozygotes is extremely rare and scarcely reported. We report the case of an asymptomatic Sinhalese man, previously unscreened, who was found to have elevated liver enzymes and hemoglobin in a routine medical check-up. His ferritin was 1272 (ng/ml) (22-322) with a transferrin saturation of 61% (15-50%). MRI of the abdomen for iron content revealed primary early iron deposition in the liver and pancreas with sparing of the spleen. Genetic studies detected H63D heterozygous homeostatic iron regulator (HFE) gene mutation with a normal C282Y gene. In his erythrocytosis workup, his erythropoietin level was suppressed. However, bone marrow biopsy did not reveal morphology suggestive of a clonal disorder, and he was negative for JAK2 V617F mutation, MPL gene, JAK2 exon 12 mutations, and calreticulin gene. He was diagnosed with H63D heterozygous hereditary hemochromatosis with iron overload and erythrocytosis and commenced on venesections as treatment for both conditions, with a good response. This case report highlights the rare possibility of developing clinically significant iron overload in H63D heterozygous hereditary hemochromatosis. Furthermore, several studies have reported the detection of HFE mutations in patients previously diagnosed with 'idiopathic' erythrocytosis. Hence, this case report calls attention to the need to suspect the presence of HFE gene mutations in patients with erythrocytosis with a negative workup for clonal red cell disorders.

## Introduction

Hereditary hemochromatosis is an autosomal recessive disorder that causes increased iron absorption and tissue deposition. There are two main mutations in the homeostatic iron regulator (HFE) gene implicated in developing hemochromatosis, namely, the cysteine-to-tyrosine substitution at amino acid 282 (C282Y) and histidine-to-aspartic acid substitution at 63 (H63D) [1]. HFE gene mutations increase iron absorption in the small intestine by reducing hepcidin expression, a hormone that inhibits iron absorption. This increases the activity of iron transporters, such as ferroportin, facilitating iron absorption and predisposing to iron overload [2].

A study analyzing the frequencies of HFE genotypes in different countries reveals a prevalence of C282Y homozygosity, C282Y heterozygosity, H63D heterozygosity, and H63D homozygosity of 0%, 0%, 16.5%, and 0.9%, respectively in Sri Lanka, in contrast to the global prevalence of 0.4%, 7.8%, 19.4%, and 1.9%, respectively [3].

C282Y homozygosity carries the most significant risk of iron overload [4]. C282Y:H63D compound heterozygotes, C282Y heterozygotes and H63D homozygotes have a much lower risk of developing iron overload. Iron overload in H63D heterozygotes is rare, reported in a few case reports, and its causal relationship role is controversial [4,5].

There have also been several studies reporting the detection of HFE mutations in patients who have been previously diagnosed with ‘idiopathic’ erythrocytosis [6-8]. We report the case of a 36-year-old man who was incidentally detected to have high liver enzymes and hemoglobin, which culminated in a diagnosis of hereditary hemochromatosis with H63D heterozygosity, developing clinically significant iron overload with concomitant erythrocytosis.

## Case presentation

A 36-year-old Sinhalese man was admitted to a tertiary care center in Sri Lanka for the evaluation of incidentally detected elevated liver enzymes and hemoglobin levels during a medical check-up. He was asymptomatic with no abdominal pain, fever, jaundice, pale stools, dark urine, or pruritus. He also did not have any headaches, visual disturbances, or dizziness. Furthermore, he denied any skin pigmentation, joint pain, fatigue, exertional dyspnea, chest pain, or erectile dysfunction to suggest any organ involvement due to iron deposition. He had no past medical history of note and no previous surgeries. There was no family history of liver disease in his parents or siblings. However, none had been screened for hereditary hemochromatosis. He was a non-smoker and consumed alcohol infrequently. He had no history of allergies.

On examination, he had a BMI of 25.5 kg/m^2^, was not pale or icteric, and had no stigmata of liver disease. His cardiovascular, respiratory, abdominal, and neurology examinations were unremarkable. His percentage oxygen saturation was normal at 99% on room air. A summary of his basic investigations is depicted in Table 1.

**Table 1:** Summary of basic investigations. LDL: low-density lipoprotein, HDL: high-density lipoprotein.

Test	Result	Normal range
White blood cell count (x10^3^/uL)	7.05	4.0-10.0
Hemoglobin (g/dL)	18.1	13-16.5
Mean corpuscular volume (MCV) (fL)	91.0	80-100
Mean corpuscular hemoglobin (pg)	31.2	27-34
Mean corpuscular hemoglobin concentration (g/L)	342	320-360
Red blood cell count (x10^6^/uL)	5.65	3.5-5.5
Hematocrit (%)	54	37-54
Platelet count (x10^3^/uL)	170	150-450
Aspartate aminotransferase (U/L)	422	0-50
Alanine aminotransferase (U/L)	371	0-55
Total bilirubin (mg/dl)	2.0	0-0.5
Direct bilirubin (mg/dl)	0.6	0-0.2
Serum alkaline phosphatase (U/L)	235	100-290
Serum gamma-glutamyl transferase (GT) (U/L)	494.5	0.1-49
Serum albumin (g/L)	42.4	35-50
Serum globulin (g/L)	34.6	25-35
Ferritin (ng/ml)	1272	22-322
Serum iron (ug/dL)	211	65-175
Transferrin saturation (%)	61	15-50%
Total iron binding capacity (ug/dL)	345	255-450
Serum sodium (mmol/L)	138	135-145
Serum potassium (mmol/L)	3.6	3.5-5.5
Serum creatinine (mg/dl)	0.8	<1.2
Prothrombin time (s)	15.0	11.5-15
International normalized ratio	1.0	<1.4
Erythrocyte sedimentation rate (mm/hr)	6	0-15
C-reactive peptide (mg/L)	5	<6
Total cholesterol (mg/dl)	348	140-239
Serum triglycerides (mg/dl)	159	10-200
HDL cholesterol (mg/dl)	66	35-85
Non-HDL cholesterol (mg/dl)	282	55-189
LDL cholesterol (mg/dl)	250	75-159
Fasting blood sugar (mg/dl)	99	<100

The blood picture revealed an increased red blood cell count with normochromic normocytic cells, concluding evidence of erythrocytosis, supported by the elevated hemoglobin level and hematocrit.

Serum erythropoietin level was suppressed at 1.47 mIU/ml (5.4-31). Genetic studies for JAK2 V617F mutation by real-time polymerase chain reaction (PCR), MPL gene, and JAK2 exon 12 mutations using the Sanger sequencing technique and calreticulin (CALR) gene insertions and deletions in exon 9 by fragment analysis technique were performed, and all were negative. Bone marrow biopsy did not reveal any morphological features suggestive of a clonal disorder of red blood cells.

**Figure 1:**
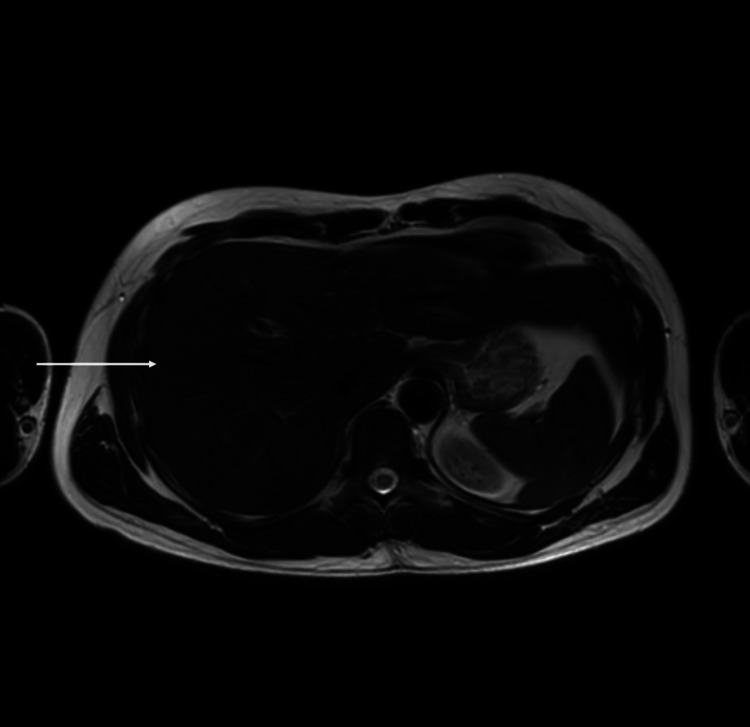
MRI of the abdomen. This study revealed mildly reduced T2 signal intensity in the liver with sparing of the spleen compatible with early primary iron deposition.

Hepatitis B surface antigen, hepatitis C IgM, IgG antibodies, and hepatitis A IgM antibodies were negative. Ultrasound (USS) abdomen revealed grade 1 fatty liver. Genetic analysis of p.H63D and p.C282Y mutations of the HFE gene revealed heterozygosity of p.H63D and normal p.C282y. An MRI of the abdomen for iron content revealed mildly reduced T2 signal intensity of the liver and pancreas without signal loss in the spleen, which is compatible with primary iron deposition in the liver and pancreas (Figure 1).

Iron estimation values showed early iron deposition in the liver with a maximum of 2.7 mg/g in segment six of the liver. He was diagnosed with hereditary hemochromatosis with H63D heterozygosity, iron overload, and erythrocytosis. The infrequent consumption of alcohol may have also contributed to the development of iron overload in this patient. He was commenced on twice weekly venesections. He was regularly followed up, and four months after diagnosis, his ferritin was 82 ng/ml (22-322) with a hemoglobin of 13.6 g/dL (13.5-16), indicating satisfactory response to venesections.

## Discussion

The most prevalent mutation in hereditary hemochromatosis patients changes the 282 cysteine residue to tyrosine (C282Y). The role of a second mutation that changes the 63 histidine-to-aspartic acid (H63D) in iron overload has been controversial. Iron overload in hereditary hemochromatosis is mainly seen with C282Y homozygosity, and in H63D heterozygotes, it is rare and scarcely reported. Two cases of H63D heterozygosity causing iron overload in the absence of other predisposing factors have been reported. In these cases, severe hemochromatosis with liver cirrhosis, diabetes, osteoporosis, and hypogonadism was noted. However, the severity of the disease in these reports highlighted the need to search for other gene mutations involved in iron metabolism, postulating the likely presence of an additional gene mutation causing iron overload in these patients [9].

A study done using the pooled analysis of 14 case-control studies revealed that C282Y homozygosity carried the most risk of iron overload, followed by C282Y/H63D compound heterozygotes, H63D homozygotes, and C282Y heterozygotes. The odds ratio for H63D heterozygotes developing iron overload was 1.6 with a 95% confidence interval of 1.0-2.6, hence yielding the association uncertain [4].

Gochee et al. assessed the role of H63D mutation in body iron metabolism by studying 62 individuals with homozygous H63D mutation, 711 heterozygotes, and 1758 controls. It was found that the presence of the H63D mutation was associated with a significant increase in serum transferrin saturation but did not cause significant iron overload in both homozygotes and heterozygotes. Furthermore, it was concluded that with the absence of the C282Y mutation, the H63D mutation is not clinically significant [10].

Studies show that even H63D homozygotes have a reduced likelihood of developing iron overload. A retrospective analysis of 170 H63D homozygous individuals also found that only 6.7% had iron overload after follow-up [1]. Another study was conducted to assess whether the coexistence of other genes would contribute to the development of iron overload in H63D homozygous patients. Forty-five H63D homozygotes were recruited, and no additional gene mutations were found. Therefore, it was concluded that, albeit rare, H63D homozygosity could be a cause of primary iron overload [11]. These studies further reiterate the rarity of iron overload in patients with H63D mutations.

There are reports that iron intake, excessive alcohol use, smoking, and male sex were positively associated with developing iron overload in patients with HFE mutations [12]. Several case reports have demonstrated the presence of comorbid conditions that may push an H63D heterozygote into iron overload. Such a report describes a patient with heterozygous H63D mutation developing clinically significant iron overload in the background of hepatitis C. This case also highlights the fact that heterozygous H63D carriers do not usually develop iron overload. Iron overload has been reported to occur in hepatitis C patients; however, it has been reported to a mild degree, suggesting that the presence of H63D heterozygosity may be a risk factor for developing clinically significant iron overload [13].

Melis et al. evaluated the effect of the H63D mutation on the ferritin levels of beta-thalassemia carriers and reported that beta-thalassemia carriers with H63D homozygous mutation have higher ferritin levels. It was postulated that the H63D mutation may be exerting a modulating effect on iron absorption [5]. Another case report describes a 64-year-old man diagnosed with hereditary spherocytosis presenting with cirrhosis with markedly high serum ferritin and transferrin saturation. This case highlighted that hemolysis alone in hereditary spherocytosis does not usually result in clinically significant iron overload and that the presence of an H63D heterozygous mutation increases the risk of iron overload development [14].

There are very few case reports of H63D mutation causing significant iron overload. Parkash et al. describe an atypical presentation of hereditary hemochromatosis in an 89-year-old man with heterozygous H63D mutation. This case highlighted that heterozygous C282Y carriers with iron overload have other conditions, such as alcohol use or liver disease, which predispose them to iron overload, and the lack of such literature for heterozygous H63D carriers [15].

In this case, the index patient was also found to have a persistently high hemoglobin level with suppressed erythropoietin level despite a negative panel for mutations of myeloproliferative disease and absent bone marrow biopsy evidence of clonal red cell disorder. The earliest description of the association between hemochromatosis and erythrocytosis was reported in 1958, in a case series, which reported erythrocytosis in patients with hereditary hemochromatosis with hepatoma [16].

A study conducted to determine hemoglobin levels in patients with hemochromatosis observed that in patients with at least one HFE mutation, higher median Hgb levels were noted, and anemia was not seen in any of the 152 patients with HFE mutations. Median hemoglobin was noted to be 15.5 g/dL, 16g/dL, 15.8g/dL, and 17.5g/dL for participants with homozygous C282Y mutation, homozygous H63D mutation, heterozygous for single mutation each of C282Y/H63D, and one with both H63D and S65C, respectively [17].

Burlet et al. recruited 132 patients diagnosed with 'idiopathic' erythrocytosis and tested them for HFE gene mutations. It was found that 73 (55%) had HFE gene mutations, of which 47 had H63D heterozygous mutations. This study concluded that HFE mutations are frequently observed in 'idiopathic' erythrocytosis and may facilitate an increase in the red cell mass [18]. A similar study reported 44.6% of the total 'idiopathic' erythrocytosis population having HFE gene mutations [6]. Benetti et al. studied 118 patients with erythrocytosis and found that 57.1% had HFE variants [7].

An update on the diagnosis and management of JAK2 negative erythrocytosis describes that in the presence of a negative workup for erythrocytosis, the presence of HFE mutations should be considered. It was postulated that this could be secondary to augmented iron uptake by erythroid cell precursors in the bone marrow. This indicates the need for additional studies to be pursued to explore the association between HFE mutations and erythrocytosis [8].

## Conclusions

A key point highlighted by this case report is the rare possibility of developing clinically significant iron overload in H63D heterozygous hereditary hemochromatosis. Furthermore, this case calls attention to the relationship between HFE gene mutations and erythrocytosis and the need to suspect HFE gene mutations in patients with erythrocytosis, in the presence of low erythropoietin, with a negative workup for clonal red cell disorders. Further studies are required to assess the role of H63D heterozygous mutation in causing clinically significant iron overload and the possible causal relationship with erythrocytosis.
